# Surgery

**Published:** 1908-09

**Authors:** T. Carwardine


### SURGERY.

On a former occasion we reviewed the progress of **gastro-enterostomy for benign conditions of the stomach and duodenum,**[3] and sufficient time has elapsed to enable conclusions to be drawn from the ultimate results of a large number of cases following operation on modern lines. The risk of operation has been reduced to a minimum; much has been learned in pathology from direct observation; and it only remains to give more atten-

[3] *Bristol M.-Chir. J.*, 1906, xxiv. 254.

tion to the selection of suitable cases for relief, eliminating unsuitable ones, and to determine the amount and the permanency of the relief afforded. Such data can only be obtained from a careful study of a large number of cases after sufficient time has elapsed, and at the meeting of the American Medical Association, in May last, three surgeons of repute were able to furnish the profession with such particulars.

Dr. W. J. Mayo[1] pointed out that no case was operated upon until a reasonable amount of medical treatment had failed, and that no case should be considered cured until two years had elapsed after operation. In earlier work technical errors occurred, and the operation was applied without due discrimination, and in some cases upon erroneous diagnosis, for modern surgery has shown that ulceration may be absent although diagnosed clinically. Dr. Mayo and his brother have operated on 540 cases of ulcer of the stomach and duodenum. Of twenty-seven cases of acute perforation, in five primary gastro-enterostomy was done, with two deaths ; but out of eighteen which recovered after suture and drainage, only one required a secondary gastro-enterostomy.

Before the year 1900 an anterior anastomosis was done, with a mortality of 6 per cent., and many have remained well from eight to fifteen years. From 1900 to 1905 operation was extended to cases without obstruction, but the results were not so favourable, owing to technical imperfections and to the fact that experience shows that it is best to excise gastric ulcers occurring to the left of the pyloric end. At this time the posterior " no-loop " operation became the method of choice, and three hundred of such operations have been performed, with a mortality under 1 per cent., and only 1 per cent. of secondary operations have been required. At this period many operations were done on the clinical diagnosis of ulcer rather than on the operative, the operation as well as the diagnosis proving to be a mistake.

Since 1905, if the ulcer could not be demonstrated at the time of exploration, no operation was done unless necessitated for hemorrhage. Three hundred and seventy-nine cases were operated upon two years ago, with a mortality of 4.8 per cent. In sixty-four no ulcer was actually demonstrated at the time of operation, and eleven of the sixty-four patients required secondary operations, only two of which showed that an ulcer had been overlooked at the former operation. These may be regarded as questionable cases, and, roughly speaking, one-third were cured, one-third improved, and one-third unimproved. Of 234 cases of demonstrated ulcers which were traced, over 80 per cent. were cured, 9 per cent. improved, and 4 per cent. unimproved, showing a total of about 90 per cent. cured or improved.

Dr. John B. Deaver[2] was able to trace sixty-six patients on whom he had performed operations for benign disease. Forty-

[1] *Ann. Surg.*, 1908, xlvii. 885. [2] *Ibid.*, 894.

four were free from all gastric symptoms, nine are greatly improved, five are unimproved, and eight have since died, giving 66 per cent. of cures and 80 per cent. greatly improved. The author goes into detail concerning the cases, and we may refer to the original article, but the following summaries may be of interest :—In ulcer of the stomach 62 per cent. were cured and 16 per cent. improved. Of twelve cases of duodenal ulcer, there were over 90 per cent. of cures ; and in stenosis of the pylorus and in gastrectasis there were some 65 per cent. of cures. The conclusions he draws from an analysis of his cases are those generally recognised by surgeons at the present time.

Though not so numerous as the cases analysed by the Mayo brothers, the tabulations of results by Moynihan are valuable from the point of view of practical completeness.[1] We may first summarise the lessons which he draws from a study of them. 1. The operative treatment of stomach disorders should be confined to those cases in which an organic lesion can be demonstrated at the time of operation ; and operation should not be performed as a rule if no lesion can be found, but one must regard the case as a diagnostic error. 2. In case of acute perforating ulcer, the perforation should be closed or the ulcer excised. When the ulcer is prepyloric, pyloric, or duodenal, gastro-enterostomy should also be performed. 3. If an ulcer be placed on the lesser curvature at some distance from the pylorus, in such a position that no obstruction is offered to the onward passage of food, excision should be performed, for relief by gastro-enterostomy may be incomplete. 4. If the ulcer be prepyloric, pyloric, or duodenal, gastro-enterostomy should be performed. It is desirable also to infold an ulcer whenever possible, for both hemorrhage and perforation have occurred from ulcers for which gastro-enterostomy had been performed months or years before. 5. The most satisfactory method of gastro-enterostomy is the posterior "no-loop" operation, with the almost vertical application of the bowel to the stomach according to his view. A deviation to one side or the other is of no importance, and entails no untoward consequences. 6. Regurgitant vomiting occurs as the result of the "loop" operation, whether anterior or posterior, and can be effectively cured by entero-anastomosis. 7. In cases of "hour-glass stomach" double operations are frequently necessary. . . . There were twenty-seven cases of perforating ulcer of the stomach and duodenum, of which eighteen recovered ; in six cases gastro-enterostomy was simultaneously performed, and in two cases subsequently, *i.e.* in eight out of eighteen. The cases suggest the desirability of gastro-enterostomy being performed when the ulcer is at or near the pylorus, and of excision of the ulcer when it is near the lesser curvature and away from the pylorus.

[1] *Ibid.*, 873.

For acute hemorrhage twenty-seven patients were submitted to operation, with two deaths. Reports have been received from twenty-two—eighteen were regarded as absolutely cured, one was improved but delicate, and three had bilious vomiting afterwards.

Of gastric and duodenal ulcers there were 174 cases, the ulcer being demonstrated in each. Of eleven cases in which no demonstrable ulcer was found, only three are described as being cured, one died from uræmia, six are no better, and in three improvement is doubtful. It is interesting to note that all the three cases on which pyloroplasty was performed required gastro-enterostomy subsequently. Post-operative vomiting occurred in twenty-two cases, and post-operative hæmatemesis in two cases. In twelve cases the patients are reported to be no better, six of them having no demonstrable lesion at the time of operation. In five cases occasional vomiting occurred associated with indiscretions in diet. In ten cases the improvement was doubtful, the majority of them having occasional vomiting, and resorting to medical advice at times. They comprise cases in which the ulcer was at some distance from the pylorus. In seven cases death occurred from malignant disease of the stomach at the site of the ulcer, all within four years of the time of operation. There were twenty-two cases of "hour-glass stomach," with three deaths, seventeen known to be alive and well.

The ultimate results have proved to be as follows:—211 patients are cured, nine patients are improved, twelve are no better, nine are doubtful, and six not reported. Thirty-four patients are dead: eighteen as the result of operation, seven of carcinoma of the stomach, and nine from causes unconnected with the stomach or operation.

From the foregoing it will be seen that much has been already accomplished, and that a high percentage of success follows the surgical treatment of benign conditions of the stomach and duodenum which have resisted a reasonable amount of medical treatment. In a considerable proportion of cases of cancer of the stomach there is a distinct history of previous gastric ulceration, and it would be interesting, as well as a valuable indication in treatment, to know what proportion of simple ulcers eventually become malignant.

T. CARWARDINE.

